# Cocaine Users Manifest Impaired Prosodic and Cross-Modal Emotion Processing

**DOI:** 10.3389/fpsyt.2013.00098

**Published:** 2013-09-05

**Authors:** Lea M. Hulka, Katrin H. Preller, Matthias Vonmoos, Sarah D. Broicher, Boris B. Quednow

**Affiliations:** ^1^Department of Psychiatry, Psychotherapy, and Psychosomatics, Experimental and Clinical Pharmacopsychology, University Hospital of Psychiatry Zurich, Zurich, Switzerland; ^2^Department of Neurology, University Hospital Zurich, Zurich, Switzerland

**Keywords:** cocaine, addiction, drug dependence, social cognition, emotion recognition, emotion perception, prosody, cognitive empathy

## Abstract

**Background:** A small number of previous studies have provided evidence that cocaine users (CU) exhibit impairments in complex social cognition tasks, while the more basic facial emotion recognition is widely unaffected. However, prosody and cross-modal emotion processing has not been systematically investigated in CU so far. Therefore, the aim of the present study was to assess complex multisensory emotion processing in CU in comparison to controls and to examine a potential association with drug use patterns.

**Method:** The abbreviated version of the comprehensive affect testing system (CATS-A) was used to measure emotion perception across the three channels of facial affect, prosody, and semantic content in 58 CU and 48 healthy control (HC) subjects who were matched for age, sex, verbal intelligence, and years of education.

**Results:** CU had significantly lower scores than controls in the quotient scales of “emotion recognition” and “prosody recognition” and the subtests “conflicting prosody/meaning – attend to prosody” and “match emotional prosody to emotional face” either requiring to attend to prosody or to integrate cross-modal information. In contrast, no group difference emerged for the “affect recognition quotient.” Cumulative cocaine doses and duration of cocaine use correlated negatively with emotion processing.

**Conclusion:** CU show impaired cross-modal integration of different emotion processing channels particularly with regard to prosody, whereas more basic aspects of emotion processing such as facial affect perception are comparable to the performance of HC.

## Introduction

Cocaine addiction continues to be a major public health concern owing to its globally widespread use (1) and the resultant high socioeconomic costs (2, 3). Apart from the financial burden for society, cocaine addiction also exerts debilitating effects on the individuals’ social relationships (4). It has been postulated that cocaine-induced neuroadaptations in mesocorticolimbic brain circuits are the reason, why dependent cocaine users (CU) attribute excessive salience to drugs and may undertake extreme measures to procure the drug, while at the same time neglecting their social duties and putting less effort and value into social interactions (4–6). Accumulating evidence from neuroimaging studies suggests that chronic CU exhibit marked structural and functional alterations in brain regions that are crucially involved in orchestrating social cognition including the orbitofrontal cortex (OFC), medioprefrontal cortex (MPFC), anterior cingulate cortex (ACC), striatum, and the temporal cortices embodying the amygdalae and insulae (4, 6–13).

Essentially, social cognition refers to a person’s ability to infer and understand another person’s state of mind but also comprises more basic aspects such as facial emotion recognition (7, 12, 14, 15). Processing of emotional information plays a fundamental role in the evaluation of social interactions and consequently shapes adequate decisions and behavioral responses in everyday life situations. Accordingly, social cognition has been associated with the development, course, and outcome of psychiatric disorders (14, 16) and is likely to influence the course of dependence and treatment success in stimulant users (4, 17). Furthermore, individuals with impaired social cognitive abilities and troubled interpersonal relationships may receive less social support, a factor that is strongly associated with more successful abstinence (18).

To date, a small but increasing number of studies have investigated different aspects of social cognition in CU (19–23). The majority of these studies have indicated that non-dependent and dependent CU (according to the DSM-IV criteria) are able to adequately recognize most of the basic emotions expressed in faces [(20); with exception of fear; (21–23)], but that dependent CU show impaired performance with regard to mental perspective taking and higher-level emotional reasoning such as understanding, managing, and regulating emotions, especially after longer duration and higher cumulative doses of cocaine use (19, 20, 23). Moreover, a study using complex and ecologically valid stimulus material more likely reflecting real-life situations also provided evidence that non-dependent and dependent CU showed diminished explicit and implicit emotional empathy (23). Interestingly, in this study impaired emotional empathy of CU was associated with a smaller social network size and a higher number of committed criminal offenses (23). Finally, in a social interaction paradigm, CU distributed money in a more self-serving manner and thus acted less altruistic compared to drug-naïve healthy controls (HCs) (24). Altogether, these findings suggest that while the more basic social cognitive abilities are preserved in CU, more complex aspects of social cognition, likely requiring the simultaneous integration of different stimulus modalities, appear to be specifically impaired in dependent CU. Despite the importance of simultaneously integrating visual and auditory information during social interactions, to date multisensory emotion processing has not been systematically investigated in CU. However, obtaining a more sophisticated understanding regarding which aspects of complex social cognitive abilities are compromised in CU might provide a unique opportunity to conceptualize more effective treatment strategies.

Therefore, the primary goal of the present study was to investigate, if, and to what extent, CU show impaired processing of complex social stimuli in comparison to HC. For this purpose, participants completed the abbreviated version of the comprehensive affect testing system (CATS-A) (25). The CATS-A enables the measurement of multisensory integration of facial and vocal affect perception across the three channels of facial affect, prosody, and semantic content.

As prior studies have shown that CU exhibited more clinically relevant symptoms and impaired performance on neuropsychological measures (24, 26, 27), a secondary aim was to examine potential associations of demographic variables, psychopathology, real-life social functioning, and cognitive parameters with emotion processing in CU and HC. Furthermore, associations with drug use patterns were examined to detect possible dose-dependent drug effects on emotion recognition. We expected that CU would exhibit an inferior performance compared to HC specifically with regard to more complex social cognitive stimuli requiring multisensory integration but not basic facial affect recognition.

## Materials and Methods

### Participants

The final study sample comprised of 48 drug-naïve HC and 58 CU selected from the second assessment of the longitudinal Zurich Cocaine Cognition Study (ZuCo^2^St) (23, 24, 26, 27). Participants were recruited through drug prevention and treatment centers, psychiatric hospitals, advertisements in local newspapers, internet platforms, and word-of-mouth communication. Inclusion criteria for all participants were: (1) age between 18 and 60 years, (2) proficiency in German language, (3) no use of prescription drugs affecting the CNS, (4) no current or previous Axis I DSM-IV psychiatric disorder (in CU with exception of cocaine abuse/dependence and/or alcohol and nicotine abuse/dependence, attention deficit hyperactivity disorder, and a history of depression), (5) no neurological disorder or head injury, (6) no family history of a severe DSM-IV psychiatric disorder such as schizophrenia, bipolar disorder, or obsessive-compulsive disorder. CU either had to meet the DSM-IV criteria for cocaine abuse or dependence (28). In order to exclude participants with opioid use and/or pronounced poly-toxic drug use patterns and to objectively characterize drug use over the past 6 months, hair samples (6 cm) from all participants were collected and analyzed with liquid chromatography-mass spectrometry (for details see Appendix). Moreover, participants were instructed to abstain from illegal drugs for ≥3 days and ≥24 h from alcohol. Urine toxicology analyses were performed to control for recent drug use (for details see Appendix). Additionally, HC were excluded if they regularly engaged in illegal drug use (>15 occasions) with the exception of cannabis use. The study was approved by the Ethics Committee of the Canton Zurich and the Swiss Federal Office of Public Health. All participants provided written informed-consent and were compensated for their participation.

Initially, CATS-A data were available for 141 subjects but 35 participants were excluded from the analysis because of the following reasons: insufficient German knowledge (1 HC), poly-toxic drug use patterns (14 CU), incongruent self-report and hair toxicology results (5 CU), history of apoplexy (1 CU), and regular intake of benzodiazepines or antidepressant drugs (6 CU). Finally, for matching reasons eight HC with the highest verbal intelligence quotients (IQ) were blindly excluded.

### Clinical interviews and questionnaires

All participants completed the *Structured Clinical Interview for DSM-IV Disorders* (SCID-I), which was carried out by a trained psychologist (29) and the DSM-IV *self-rating questionnaire for axis-II personality disorders* (SCID-II) (30). Drug use patterns were assessed by means of the *Interview for Psychotropic Drug Consumption* (IPDC), which has been described in detail elsewhere (31). Premorbid verbal IQ was estimated with the *Mehrfachwahl–Wortschatz-Intelligenztest* (MWT-B) (32). The brief version of the *Cocaine Craving Questionnaire* (CCQ) was used to assess current cocaine craving in CU (33, 34). The *Fagerström Test for Nicotine Dependence* (FTND) was used to determine the severity of nicotine dependence (35). Current symptoms of depression were measured with the *Beck Depression Inventory* (BDI) (36). In order to assess a real-world correlate of social functioning, the *Social Network Questionnaire* (SNQ) was applied, which is based on the social contact circle interview (37). The SNQ assesses the size of an individual’s social network in different areas such as “household,” “family,” “work or apprenticeship,” “friends,” “neighbors,” “(sport) clubs or unions,” and “others.” Only individuals with whom the participants have had contact over the past 4 weeks counted as personal contacts and double entries in different areas were not allowed to enable the calculation of the total social network size. In the second test assessment of the ZuCo^2^St study, the SNQ layout was slightly restructured. Therefore, the numbers of the total social network size differ between the first and second assessment. Furthermore, participants underwent a broad neuropsychological and social cognitive test battery as well as psychophysiological measurements, which have been described in detail elsewhere (23, 24, 26, 27).

### Comprehensive affect testing system

The CATS-A is an abbreviated, computerized version of the comprehensive affect testing system (25, 38). The test comprises of visual (Ekman basic emotions) and auditory stimulus material either requiring facial affect recognition, identification of non-emotional and emotional prosodic information, as well as simultaneous cross-modal processing of conflicting prosody and lexical content or distinguishing of incongruent facial expressions and prosodic information. By means of the CATS-A it is not only possible to determine if a person exhibits a general deficit in emotion perception, but also if one of the channels is more compromised. The performance for each item is either scored as correct or incorrect and subsequently the sum and/or the percentage of correct items can be calculated for each subtest. Two validity scales, comprised of items found to be very easy for most HC subjects, are embedded in the test to detect poor effort. The time to complete the CATS-A ranged from 30 to 40 min.

#### Subtests

The CATS-A is composed of 13 subtests: (1) identity discrimination, (2) affect discrimination, (3) non-emotional prosody discrimination, (4) emotional prosody discrimination, (5) name affect, (6) name emotional prosody, (7) match affect, (8) select affect, (9) conflicting prosody – attend to prosody, (10) conflicting prosody – attend to meaning, (11) match emotional prosody to emotional face, (12) match emotional face to emotional prosody, (13) Three Faces Test. A detailed description can be found in the CATS manual (39).

#### Quotient scales

The emotion recognition quotient (ERQ) is comprised of all 11 emotion subtests (2, 4–13) and excludes the non-emotional subtests 1 and 3. The ERQ determines whether an overall impairment in emotion recognition is present. The affect recognition quotient (ARQ) and the prosody recognition quotient (PRQ), comprising of the subtests 2, 5, 7, 8, 13 and 4, 6, 9 respectively, enable to detect if one of the communication channels is more affected.

#### Composite scales

The Simple Facial Scale includes the subtests 2 and 5, the Complex Facial Scale includes subtests 7, 8, and 13, the Prosody Scale is equivalent to the PRQ and includes subtests 4, 6, and 9, the Lexical Scale equals subtest 10, and the Cross-Modal Scale is comprised of subtests 11 and 12.

#### Discrete emotion scales

The discrete emotion scales consist of facial expression images depicting the six basic emotions “happiness,” “surprise,” “fear,” “sadness,” “anger,” and “disgust” and are derived from the subtests 5, 7, 8, and 13.

### Cognition

The Letter Number Sequencing Task (LNST) (40) was used to measure working memory function. The dependent variable was the number of correct answers. The Rapid Visual Processing task (RVP) from the Cambridge Neuropsychological Test Automated Battery (CANTAB) was administered to assess sustained attention (www.cantab.com). The dependent variable was A’, a signal detection measure of sensitivity that incorporates how well a person is able to detect target sequences.

### Statistical analysis

Statistical analyses were performed with the IBM SPSS Statistics 19.0 software (SPSS Inc., Zurich). Independent Student’s *t*-tests and frequency analyses (Pearson’s Chi^2^-test) were conducted to determine if groups differed with regard to demographic variables, questionnaire data, and CATS-A measures. To account for multiple testing, group comparisons for the CATS-A were Bonferroni-corrected, resulting in a significance level of *p* < 0.0167 (*p* = 0.05/3) for the quotient scales, *p* < 0.0038 (*p* = 0.05/13) for the subtests, and *p* < 0.0083 (*p* = 0.05/6) for the discrete emotion scales. Pearson’s product-moment correlations were used to examine if the performance on the CATS-A measures was associated with demographic variables, questionnaire data, and drug use parameters. To avoid alpha-error accumulation but to enable the detection of moderate correlations, the significance level in correlation analyses was set at *p* < 0.01. The potential association of co-factors such as sex, recreational cocaine use vs. cocaine dependence, residual effects of cannabis and cocaine, and cognition with the performance on the CATS-A was assessed with analyses of variance (ANOVA), analysis of covariance (ANCOVA), and multiple regression analyses. Effect sizes (Cohen’s *d*) were calculated with G*Power 3.1 (41). Due to non-parametric distributions, the drug use parameters weekly alcohol, cigarette, cannabis, and cocaine consumption (*g* or number of cigarettes), last alcohol, cigarette, cannabis, and cocaine use (abstinence duration in days), cumulative cannabis and cocaine dose (*g*), and concentrations of cocaine and its metabolites in the hair samples (picogram per milligram) were log-transformed (log10) and the constant 1 was added because the data contained 0 values.

## Results

### Demographic information, self-report questionnaires, and drug use patterns

HC and CU did not differ with regard to age, sex distribution, years of education, verbal IQ, and smoking status (Table 1). CU scored significantly higher on the BDI and FTND compared to HC indicating that CU reported more symptoms of depression and more severe nicotine dependence. As expected and consistent with the results from the baseline assessment of the present study (23), CU scored higher on the antisocial personality disorder (PD) and the narcissistic PD scale of the SCID-II, and had a smaller overall social network size than HC. Eighteen CU (31%) met the DSM-IV criteria for cocaine dependence. Two CU indicated to smoke cocaine, while all the others used cocaine nasally.

**Table 1:** **Demographic data (means and standard deviations, number of subjects, and percent)**.

	Controls (*n* = 48)	Cocaine users (*n* = 58)	Value^a^	*p*	*df*
Age	30.42 (±8.37)	32.31 (±8.44)	−1.15	0.25	104
Sex (m/f)	33, 15 (69, 31%)	46, 12 (79, 21%)	1.54^b^	0.21	1
Years of education	10.76 (±1.65)	10.37 (±1.68)	1.20	0.23	104
Verbal IQ (MWT-B)	107.02 (±9.88)	104.40 (±8.03)	1.51	0.13	104
Beck Depression Inventory (BDI)	2.65 (±4.65)	7.84 (±7.85)	−4.04	0.00	104
Narcissistic Personality Disorder Scale (SCID-II)	2.13 (±1.77)	4.00 (±2.66)	−4.02	0.00	89
Antisocial Personality Disorder Scale (SCID-II)	2.55 (±1.80)	4.55 (±3.28)	−3.73	0.00	91
Social Network Questionnaire (SNQ) (total size)	17.81 (±6.20)	13.93 (±6.78)	3.01	0.00	100
Cocaine Craving Questionnaire (CCQ) (sum)	–	17.13 (±8.90)	–	–	–
Smoking status (yes/no)	38, 10 (79, 21%)	49, 9 (84, 16%)	0.51^b^	0.48	1
Fagerström Test for Nicotine Dependence (FTND) (sum)	2.21 (±2.52)	3.60 (±2.68)	−2.43	0.02	83

*^a^Independent t-test, ^b^Chi^2^-test for frequency data*.

Drug use patterns are shown in Table 2. CU reported higher weekly alcohol consumption [*t*(104) = −2.99, *p* < 0.01], indicated to have used cannabis for a longer duration [*t*(104) = −2.69, *p* < 0.01], and more frequently tested positive for cannabis in the urine toxicology [*X*^2^(1) = 6.32, *p* < 0.05] than HC. While HC did not use cocaine, amphetamine, and MDMA at all, CU reported an average weekly cocaine consumption of 0.93 g, relatively little co-use of amphetamine and MDMA, and no use of methamphetamine and opiates, which was confirmed by the hair toxicology analyses. Given that some drug urine screenings were positive additional analyses were carried out to investigate the effects of recent cocaine and cannabis use.

**Table 2:** **Drug use patterns (means and standard deviations)**.

	Controls (*n* = 48)	Cocaine users (*n* = 58)
**ALCOHOL**		
Grams per week	95.33 (±81.28)	185.83 (±195.79)**
Years of use	12.91 (±8.48)	13.91 (±7.84)
**NICOTINE**		
Cigarettes per week	58.68 (±57.98)	75.24 (±69.51)
Years of use	9.89 (±8.74)	11.44 (±8.74)
Last consumption (days)	2.28 (±6.17) (*n* = 38)	2.82 (± 11.57) (*n* = 49)
**CANNABIS**		
Grams per week	0.52 (±1.57)	1.41 (±3.22)
Years of use	4.34 (±5.63)	8.31 (±8.88)**
Cumulative dose (g)	1005.42 (±3993.87)	2484.93 (±5465.40)
Last consumption (days)	158.79 (±535.18) (*n* = 26)	41.21 (±74.51) (*n* = 36)
Urine toxicology (pos./neg.)	7, 41 (15, 85%)	21, 37 (36, 64%)*
**COCAINE**		
Times per week	0.00 (±0.00)	0.67 (±0.76)***
Grams per week	0.00 (±0.00)	0.93 (±1.68)***
Years of use	0.00 (±0.00)	8.60 (±5.55)***
Maximum dose (g/day)	–	3.16 (±2.81)
Cumulative dose (g)	0.00 (±0.00)	1901.77 (±5110.32)*
Last consumption (days)	–	40.36 (±91.14)
Cocaine in hair (pg/mg)	0.00 (±0.00)	14620 (±35632)**
Benzoylecgonine in hair (pg/mg)	0.00 (±0.00)	3610 (±11713)*
Ethylcocaine in hair (pg/mg)	0.00 (±0.00)	779 (±1826)**
Norcocaine in hair (pg/mg)	0.00 (±0.00)	327 (±872)*
Urine toxicology (pos./neg.)	0, 100 (0, 100%)	17, 41 (29, 71%)***
**AMPHETAMINE**		
Grams per week	0.00 (±0.00)	0.05 (±0.15)*
Years of use	0.00 (±0.00)	2.23 (±4.43)**
Cumulative dose (g)	0.00 (±0.00)	27.01 (±104.53)
Last consumption (days)	–	116.65 (±104.63) (*n* = 19)
Amphetamine in hair (pg/mg)	0.00 (±0.00)	46 (±158)*
Methamphetamine in hair (pg/mg)	0.00 (±0.00)	0.00 (±0.00)
**MDMA**		
Pills per week	0.00 (±0.00)	0.14 (±0.53)
Years of use	0.00 (±0.00)	3.33 (±5.32)***
Cumulative dose (pills)	0.00 (±0.00)	60.07 (±161.23)*
Last consumption (days)	–	107.49 (±99.32) (*n* = 25)
MDMA in hair (pg/mg)	0.00 (±0.00)	605 (±2652)
MDEA in hair (pg/mg)	0.00 (±0.00)	3 (±24)
MDA in hair (pg/mg)	0.00 (±0.00)	28 (±145)

Independent t-test or Chi^2^-Test for frequency data. *p < 0.05, **p < 0.01, ***p < 0.001. Consumption per week captures the last 6 months, duration of use, and cumulative dose are averaged within the total group. Last consumption is averaged only for subjects who used the drug in the last 6 months. In this case, sample size n is shown. The hair analysis was performed on two hair samples (each 3 cm in length) per participant capturing drug use over the last 6 months. Concentrations were averaged over the two samples. If the hair sample was not long enough, only one sample was analyzed (3 cm, 3 months). MDMA, 3,4-methylenedioxy-N-methylamphetamine, methylenedioxymethamphetamine; MDEA, methylenedioxyethylamphetamine; MDA, 3,4-methylenedioxyamphetamine.

### Comprehensive affect testing system

Initial analysis of the validity scales *V*_1_ + *V*_2_ and (*V*_1_-*V*_2_) confirmed that all participants showed adequate levels of effort during test performance.

#### Quotient scales

Cocaine users had a significantly lower ERQ and PRQ but not ARQ than HC, indicating that although CU performed worse in general emotion recognition, they were specifically impaired in processing prosodic information but not affect recognition (Table 3). Results remained significant even after applying a Bonferroni correction.

**Table 3:** **Independent *t*-tests of the comprehensive affect testing system (CATS-A) for controls and cocaine users (means and standard deviations) of correct responses in percentage**.

	Controls (*n* = 48)	Cocaine users (*n* = 58)	*t*-Value	df	*p* Value	Cohen’s *d*
**QUOTIENTS**						
Emotion recognition quotient (ERQ, items 2, 4–13)	78.79 (±7.27)	74.63 (±8.10)	2.75	104	**0.007**	**0.53**
Affect recognition quotient (ARQ, items 2, 5, 7, 8, 13)	76.04 (±9.09)	73.51 (±10.07)	1.35	104	0.180	0.26
Prosody recognition quotient (PRQ, items 4, 6, 9)	85.42 (±7.89)	78.79 (±10.41)	3.63	104	**0.000**	**0.67**
**COMPOSITE SCALES**						
Simple Facial Scale (items 2, 5)	79.51 (±11.53)	77.3 (±12.28)	0.95	104	0.344	0.18
Complex Facial Scale (items 7, 8, 13)	74.55 (±9.22)	72.29 (±10.81)	1.15	104	0.255	0.23
Prosody Scale (items 4, 6, 9)	85.42 (±7.89)	78.79 (±10.41)	3.63	104	**0.000**	**0.67**
Lexical Scale (item 10)	72.57 (±16.66)	66.67 (±18.60)	1.70	104	0.091	0.32
Cross-Modal Scale (items 10, 12)	80.47 (±10.07)	76.29 (±11.87)	1.93	104	0.056	0.37
**INDIVIDUAL SUBTESTS**						
Identity discrimination	98.61 (±5.25)	97.84 (±4.57)	0.80	104	0.424	0.14
Affect discrimination	90.45 (±11.53)	89.51 (±11.32)	0.42	104	0.674	0.09
Non-emotional prosody discrimination	90.63 (±14.14)	89.66 (±15.24)	0.34	104	0.737	0.08
Emotional prosody discrimination	98.26 (±6.19)	96.84 (±7.29)	1.07	104	0.287	0.15
Name affect	57.64 (±21.73)	52.87 (±21.88)	1.12	104	0.266	0.20
Identify emotional prosody	73.78 (±14.38)	68.25 (±17.27)	1.77	104	0.080	0.37
Match affect	78.3 (±10.97)	74.43 (±14.38)	1.53	104	0.128	0.29
Select affect	94.1 (±10.02)	95.4 (±8.72)	−0.72	104	0.475	0.13
Conflicting prosody/meaning –attend to prosody	90.63 (±10.54)	80.32 (±12.26)	4.59	104	**0.000**	**0.81**
Conflicting prosody/meaning –attend to meaning	72.57 (±16.66)	66.67 (±18.60)	1.70	104	0.091	0.32
Match emotional prosody to emotional face	72.57 (±18.03)	65.09 (±19.21)	2.05	104	**0.043**	**0.42**
Match emotional face to emotional prosody	88.37 (±10.84)	85.92 (±12.31)	1.08	104	0.285	0.22
Three faces test	67.80 (±13.03)	65.45 (±13.18)	0.92	104	0.360	0.19
**DISCRETE EMOTIONS**						
Happiness	98.96 (±3.49)	99.57 (±2.30)	−1.04	104	0.283	0.21
Surprise	82.81 (±14.95)	78.66 (±15.36)	1.40	104	0.164	0.27
Fear	83.59 (±16.54)	78.88 (±19.62)	1.32	104	0.189	0.26
Sadness	77.86 (±19.68)	75.43 (±18.28)	0.66	104	0.511	0.13
Anger	51.04 (±20.76)	47.84 (±22.35)	0.76	104	0.451	0.15
Disgust	65.1 (±21.57)	63.38 (±18.12)	0.45	104	0.657	0.09

*Statistically significant test values (p < 0.05) are depicted in bold type*.

#### Composite scales

Cocaine users scored significantly lower only on the Prosody Scale and by trend also on the Cross-Modal Scale (*p* = 0.088) compared to HC (Table 3). The effect in the Prosody Scale was significant even after correction for multiple comparisons.

#### Subtests

Cocaine users scored significantly lower on the subtests “conflicting prosody/meaning – attend to prosody” and “match emotional prosody to emotional face” in comparison to HC (Table 3), reflecting specific deficits in the processing of conflicting and multisensory information. Only the subtest “conflicting prosody/meaning – attend to prosody” survived the Bonferroni correction and CU and HC did not differ on the remaining 11 subtests.

#### Discrete emotion scales

The performance of CU and HC did not differ with regard to the discrete emotion scales, indicating that CU were not impaired in basic facial affect recognition (Table 3). Interestingly, for both, CU and HC, it was most difficult to recognize anger in facial stimuli correctly and both groups only identified anger correctly in approximately 50% of the stimuli, whereas the happiness scale was seemingly too easy for our study population as indicated by an evident ceiling effect (>98% correct answers).

### Correlation analyses

Correlations of the CATS-A quotients with demographic variables, personality scores, clinical and real-life measures, as well as cognitive parameters are shown in Table 4. Younger age, higher verbal IQ, more years of education, a larger social network size, and higher attention scores were associated with higher ERQs and PRQs. Moreover, lower narcissistic PD scores and higher working memory scores were associated with higher PRQs. Additionally, years of education and attention correlated positively with the ARQ. No associations were found between the CATS-A quotients, symptoms of depression, and the antisocial PD score. Furthermore, in CU longer durations (*r* = −0.36, *p* < 0.01) and higher cumulative doses (*r* = −0.42, *p* < 0.01) of cocaine use were associated with lower ERQs. These correlations remained significant even after correcting for age (*r* = −0.28, *p* < 0.05; *r* = −0.36, *p* < 0.01). Notably, longer durations (*r* = −0.43, *p* < 0.01) and higher cumulative doses (*r* = −0.45, *p* < 0.01) of cocaine use were also associated with lower scores on the subtest “match emotional prosody to emotional face” (corrected for age: *r* = −0.37, *p* < 0.01; *r* = −0.39, *p* < 0.01). None of the other drug use parameters (including alcohol, nicotine, and cannabis use parameters) correlated with the CATS-A quotients.

**Table 4:** **Correlations between CATS-A quotients, demographic variables, clinical and personality scores, social network size, and cognition**.

	Emotion recognition quotient	Affect recognition quotient	Prosody recognition quotient
**DEMOGRAPHY**			
Age	−0.33		−0.35
Verbal IQ (MWT-B)	0.29		0.32
Years of education	0.32	0.32	0.30
**CLINICAL SCALES**			
Beck Depression Inventory (BDI)			
Narcissistic PD score (SCID-II)			−0.28
Antisocial PD score (SCID-II)			
**REAL-WORLD MEASURE**			
Social Network Questionnaire (SNQ)	0.31		0.30
**NEUROPSYCHOLOGY**			
Letter number sequencing task (LNST)			0.27
Rapid visual processing A’ (RVP A’)	0.38	0.28	0.32

*Correlations with p < 0.01 are shown. n = 106*.

### Investigation of co-factors

#### Sex differences

To investigate the potential effect of sex on the performance on the CATS-A, an ANOVA with the two fixed factors group (CU vs. HC) and sex (men vs. women) was carried out. Confirming the main analyses, there was a significant main effect of group for ERQ [*F*(2, 101) = 6.63, *p* < 0.05], PRQ [*F*(2, 101) = 7.80, *p* < 0.01], “conflicting prosody/meaning – attend to prosody” [*F*(2, 101) = 12.93, *p* < 0.01], and “match emotional prosody to emotional face” [*F*(2, 101) = 6.08, *p* < 0.05]. The only significant main effect of sex was found for the discrete emotion scale disgust [*F*(1, 101) = 4.19, *p* < 0.05], indicating that women were slightly better able to identify disgust compared to men. The only significant interaction effect of group × sex emerged for the discrete emotion anger [*F*(1, 101) = 5.01, *p* < 0.05]. While female HC were better in detecting anger correctly than male HC, female CU had more difficulties to identify anger correctly than male CU.

#### Recreational cocaine use vs. cocaine dependence

In order to examine if dependent CU who met the DSM-IV criteria for cocaine dependence performed worse on the CATS-A than HC and non-dependent CU, an ANCOVA with age as covariate was conducted because the dependent CU were older than HC and non-dependent CU. Results revealed a significant effect of group for the ERQ [*F*(2, 101) = 3.08, *p* < 0.05], PRQ [*F*(2, 101) = 5.75, *p* < 0.01], and “conflicting prosody/meaning – attend to prosody” [*F*(2, 101) = 9.81, *p* < 001]. Bonferroni-corrected *post hoc* analyses showed that, both, non-dependent CU (*p* < 0.01) and dependent CU (*p* = 0.05) had significantly lower PRQ scores than HC. Moreover, non-dependent CU (*p* < 0.001) and dependent CU (*p* < 0.05) achieved significantly lower scores on the subtest “conflicting prosody/meaning – attend to prosody” in comparison to controls. *Post hoc* comparisons for the ERQ were not significant. The results indicated that not only dependent CU but even non-dependent CU showed impaired processing of prosodic and multimodal information.

#### Recent drug use

The potential effect of recent cannabis use was investigated by means of an ANOVA with the fixed factors of group (CU vs. HC) and cannabis toxicology [negative (*N*_CU_ = 37, *N*_HC_ = 41) vs. positive (*N*_CU_ = 21, *N*_HC_ = 7)]. Consistent with the main analyses, there was a significant group effect for the ERQ [*F*(1, 102) = 8.07, *p* < 0.01], PRQ [*F*(1, 102) = 9.70, *p* < 0.01], and “conflicting prosody/meaning – attend to prosody” [*F*(1, 102) = 12.65, *p* < 0.01]. Neither the factor cannabis toxicology nor the interaction effect group × cannabis toxicology were statistically significant, indicating that residual effects of cannabis use were not associated with altered performance on the CATS-A.

Possible associations of recent cocaine use with the performance on the CATS-A were examined with an ANOVA where HC were compared with CU who tested negative for cocaine (*N* = 41) in the urine toxicology and CU who tested positive for cocaine (*N* = 17). In line with the main analyses, groups significantly differed with regard to the ERQ [*F*(2, 103) = 3.81, *p* < 0.05], PRQ [*F*(2, 103) = 6.73, *p* < 0.01], and “conflicting prosody/meaning – attend to prosody” [*F*(2, 103) = 10.42, *p* < 0.001]. Bonferroni-corrected *post hoc* analyses revealed that CU with a negative (*p* < 0.05) but not CU with a positive urine screen (*p* = 0.29) for cocaine achieved a lower ERQ than HC. In contrast, both, CU who tested negative (*p* < 0.01, *p* < 0.001) and CU who tested positive for cocaine (*p* < 0.05, *p* < 0.01) had a significantly lower PRQ and “conflicting prosody/meaning – attend to prosody” score than HC, suggesting that recent cocaine use did not alter processing of prosodic information.

#### Cognition and verbal IQ

In order to investigate the potential association of co-factors with the performance on the CATS-A, two multiple regression models were conducted with the common predictors age, sex, verbal IQ, the group contrast CU vs. HC, and a working memory parameter (LNST) in model 1 and an attention parameter (RVP A’) in model 2 (Table 5). In model 1 and 2, age, IQ, and the group contrast were significant predictors for the ERQ and PRQ. Moreover, with regard to the ARQ, age and IQ were significant predictors in model 1 and IQ in model 2. Sex, working memory, and attention performance were not significant predictors for the CATS-A quotients, indicating that there was no general difference in the ability of emotion and prosody recognition between men and women and that working memory and attention deficits alone could not explain the worse performance of CU on the CATS-A.

**Table 5:** **Multiple regression analyses for demographic variables, IQ, cognitive parameters, and group contrast predicting CATS-A quotients**.

	Emotion recognition quotient			Affect recognition quotient			Prosody recognition quotient		
	*B*	*SE B*	β	*B*	*SE B*	β	*B*	*SE B*	β
Constant	75.11	12.77		32.21	8.14		18.24	3.53	
Age	−0.53	0.12	−0.43***	−0.21	0.08	−0.30*	−0.14	0.03	−0.40***
Sex	0.02	2.08	0.00	0.19	1.33	0.01	0.00	0.58	0.00
IQ (MWT-B)	0.45	0.12	0.36***	0.22	0.07	0.31**	0.13	0.03	0.36***
Letter number sequencing task (LNST)	−0.17	0.33	−0.05	−0.16	0.21	−0.09	−0.01	0.09	−0.01
Controls vs. cocaine users	−4.60	1.87	−0.22*	−1.36	1.19	−0.12	−1.65	0.52	−0.28*
*R*^2^		0.32			0.15			0.35	
*F*		8.26***			3.18*			9.74***	
Constant	57.93	25.33		21.55	16.19		20.72	7.00	
Age	−0.45	0.13	−0.37**	−0.15	0.08	−0.22	−0.15	0.04	−0.42***
Sex	−0.43	2.10	−0.02	−0.13	1.35	−0.01	0.04	0.58	0.01
IQ (MWT-B)	0.40	0.12	0.32**	0.18	0.08	0.26*	0.13	0.03	0.38***
Rapid visual processing (RVP A’)	19.36	28.01	0.08	11.24	17.89	0.08	−3.25	7.74	−0.05
Controls vs. cocaine users	−4.20	1.89	−0.20*	−1.08	1.21	−0.09	−1.68	0.52	−0.29**
*R*^2^		0.32			0.15			0.36	
*F*		8.33***			3.13*			9.79***	

*p < 0.05, **p < 0.01, ***p < 0.001. B, unstandardized regression coefficient; SE, unstandardized standard error; β, standardized beta.

## Discussion

The present study assessed multisensory emotion processing in CU in comparison to HC and examined potential associations with drug use patterns. Comprehensive psychiatric diagnostics assured that participants had few psychiatric comorbidities and hair and urine toxicology analyses provided an objective characterization of drug use patterns, enabling the selection of a CU sample with relatively sparse poly-toxic drug use. Our study yielded the following key findings: (I) Overall, CU exhibited significantly inferior general emotion recognition compared to HC, which was specifically driven by deficient processing of prosodic information and to some extent insufficient processing of multimodal information but did not extend to the more basic facial affect recognition and lexical processing. (II) Longer duration of cocaine use and higher cumulative cocaine doses were associated with worse performance in emotion recognition indicating that these deficits may be partially drug-induced. (III) Deficient general emotion recognition and prosody recognition were associated with a smaller social network size. (IV) Not only dependent but even non-dependent CU showed impaired prosodic information processing and recent cannabis and cocaine use did not influence prosodic information processing substantially. Age, IQ, and cocaine use were more important in predicting the performance in general emotion and prosody recognition than sex, working memory, and attention. Altogether, our results indicate that CU were able to identify discrete facial emotions adequately, whereas their cross-modal integration of different emotion processing channels particularly with regard to prosody appears to be impaired.

The main finding of the present study that CU showed adequate perception of discrete emotions but exhibited impaired processing of more complex multimodal emotion recognition is consistent with previous studies. Accordingly, studies relying on the Emotional Facial Expression (EFE) task, the Reading the Mind in the Eyes Task (RMET), the Meyer–Salovey–Caruso Emotional Intelligence Test (MSCEIT), and the Multifaceted Empathy Test (MET) demonstrated that CU were able to infer the mental state of a person from pictures only displaying the eye region and to identify most basic facial emotions equally well as HC (20, 23, 42, 43) with the exception of a replicated finding of deficient fear recognition in the EFE (21, 22, 42, 43). The selectively impaired fear recognition of CU is in contrast to the findings from our study, but also to the findings of Fox et al. (20), and Preller et al. (23). Differences in the composition of the study samples may account for the discrepant results as all of the prior studies included cocaine-preferring poly-substance users (21, 22, 42, 43), whereas we investigated a relatively pure CU group. Moreover, previous studies did not provide objective verification of self-reported drug use, while we controlled cocaine use with hair toxicology analyses. Alternatively, it is possible that the EFE is more difficult than other facial affect perception tests because levels of the depicted emotions vary in intensity in the EFE.

Our finding that CU specifically showed impaired processing of prosodic emotional information and multimodal integration of emotion perception is in line with previous reports providing evidence for difficulties in CU with regard to higher-level emotional processing including the understanding, management, and regulation of emotions as well as mental perspective taking (*theory-of-mind*) (19, 20, 23) and neuroimaging findings demonstrating that the MPFC, which is altered in chronic CU (4, 6, 8, 9), has been associated with the integration of multimodal information (44). Notably, only dependent but not recreational CU made more errors than HC in mental perspective taking in the multisensory Movie for the Assessment of Social Cognition (MASC) (23), whereas prosodic and multimodal information processing in the CATS-A were even impaired in non-dependent CU. The MASC portrays real-world situations and dialogs that may be slightly easier to process as more contextual information is provided, while the CATS-A is more abstract and cognitively demanding as only pictures of faces but not whole persons are depicted and prosodic information is not embedded in real-life dialogs.

It is noteworthy that the deficit in prosody recognition in CU was specifically confined to the subtest requiring the processing of incongruent prosody and semantic meaning (“conflicting prosody/meaning – attend to prosody”) but did neither extend to lexical comprehension, non-emotional or emotional prosody discrimination, nor to the identification of emotional prosody. Strikingly similar results with regard to prosodic processing have been reported for schizophrenia patients and it has been suggested that due to their specific nature, the deficits may occur further downstream at the level of complex emotion comprehension (45). Moreover, results derived from human neuroimaging and brain lesion studies (25, 46) have revealed that particularly right inferior frontal and striatal regions are implicated in prosodic processing. Therefore, the notion that drug-induced neuroadaptations in frontostriatal regions in CU (6, 8–11, 13) may contribute to deficient prosody processing appears plausible. On a functional level, compromised integration of prosodic and semantic information may be particularly relevant in emotionally ambiguous social situations requiring the perception of subtle ironic or sarcastic contents (47, 48). Notably, a higher score in the subtest “conflicting prosody/meaning – attend to prosody” was associated with a larger social network size (*r* = 0.33, *p* < 0.001), supporting the assumption that social difficulties of CU may partially arise from incomplete integration of stimuli from multiple information channels.

Interestingly, CU were also impaired in the subtest “match emotional prosody to emotional face” requiring the integration of emotional facial and vocal stimuli. The fact that neither simple and complex facial affect recognition, nor identification and discrimination of prosody were compromised in CU implies that the reduced audiovisual emotion matching in CU may also reflect insufficient higher-level top-down mechanisms (49, 50). Multisensory integration is essential for real-world social situations and insufficient detection, integration, and filtering of information can hamper an adequate perception of the environment as well as socially adapted behavioral reactions (51). Therefore, enhancing multisensory emotion processing in CU may be an effective treatment strategy to improve long-term psychosocial outcomes.

In a secondary analysis, we investigated potential associations of demographic variables, clinical and real-life measures, cognitive parameters, and drug use patterns with general, facial affect, and prosodic emotion recognition. In accordance with prior studies, older age was associated with a particular decline in performance on prosody (25), whereas better attention performance correlated with more accurate general emotion, facial affect, and prosody recognition and better working memory with better prosody recognition (45). Furthermore, concordant with a previous study applying the MSCEIT (20) and the CATS-A, respectively (25), in our study, higher IQ scores were associated with better general and prosodic emotion recognition. Moreover, corroborating our finding from the first assessment of the present study sample (23), demonstrating that a smaller social network correlated with deficient emotional empathy, here, a larger total social network size was associated with better general emotion and prosody recognition. Also in line with Preller et al. (23), lower narcissistic PD scores were associated with superior prosody recognition. However, as the correlations with the PD scales and prosody recognition were relatively weak, it appears that personality traits may be more strongly related to empathy rather than emotion perception *per se* (23).

The association between longer duration of cocaine use and higher cumulative cocaine doses with poorer performance in general emotion recognition is accordant with prior reports on complex social cognitive functioning in CU (20, 23), and potentially reflects that cocaine use may indeed exert a negative effect on emotion recognition. In addition, current craving for cocaine was not significantly associated with the CATS-A performance, however, the reported craving scores were not very pronounced during study participation. Overall, duration and cumulative doses of cocaine appear to be more important for complex emotion recognition performance than abstinence duration and residual drug effects.

In order to take the influence of potential co-factors on emotion recognition performance into account, we conducted some additional analyses. Specifically, the finding that not only dependent CU but even non-dependent CU showed impaired processing of prosodic and cross-modal information is highly relevant, because it could indicate that even less frequent and quantitatively lower cocaine doses may adversely affect complex emotion processing. In order to confirm a potential causal relationship of cumulative cocaine use and deteriorating complex emotion processing, longitudinal and prospective studies are needed. Moreover, the finding that CU with a negative but not with a positive cocaine urine screen scored significantly lower on general emotion recognition than HC could indicate that current or protracted withdrawal may be more relevant regarding general emotion recognition deficits or that post-acute cocaine effects may compensate deficits to a certain extent. However, future studies should investigate this issue more systematically as it is possible that this result may have been influenced by the reduced power due to the smaller group size of the CU with a positive cocaine urine toxicology (*n* = 17). Finally, an important issue that has to be taken into account for the interpretation of the current data is the fact that prosodic and multisensory information processing requires greater working memory demand than facial affect recognition (25) and CU have been shown to exhibit particularly strong neuropsychological impairment in working memory (27, 52). To address this, we conducted a regression model and found that prosodic recognition was best predicted by age, IQ, and cocaine use, whereas sex, working memory, and attention did not explain a significant amount of variance. Therefore, these results suggest that despite the greater working memory demand during prosody recognition, working memory and attention alone are not responsible for the worse prosodic information processing performance in CU.

The present study has some limitations. First, the cross-sectional nature of the data does not permit conclusions regarding causality and, hence, despite the dose-dependent relationship of cocaine use and CATS-A performance, we cannot fully exclude the possibility that preexistent deficits may have influenced multisensory emotion recognition in CU. Second, overall, the study sample comprised more men than women. Although no significant effect of sex on the different emotion recognition channels was found, women identified the discrete emotion disgust with higher accuracy than men matching previous data in which women performed better with regard to simple and complex facial affect recognition (25). Thus, it is possible that further subtle sex differences were not detected due to insufficient statistical power. Third, diagnoses of substance use disorders were based on the DSM-IV criteria. However, the DSM-V does not distinguish between cocaine abuse and dependence anymore, but instead substance use disorders are specified by criteria for intoxication, withdrawal etc. (53). Notwithstanding this limitation, we investigated many aspects of drug use patterns including drug quantity, frequency, duration, and abstinence duration as well as urine toxicology results. Forth, CU reported a rather moderate average weekly cocaine consumption of 0.93 g. Accordingly, the present results may underestimate the occurrence of emotion recognition deficits related to heavier cocaine use.

To our knowledge, this is the first systematic investigation of multisensory emotion processing across the different channels of facial affect, prosody, and semantic content in CU and HC. In summary, this study demonstrates that non-dependent and dependent CU exhibit dose- and duration-dependent impairment in the processing of prosodic and cross-modal emotion recognition but not the more basic facial affect recognition in comparison to HC. Furthermore, deficient emotion recognition is associated with a smaller social network size, in particular for CU, possibly reflecting that subtle deficits in evaluating social stimuli in daily interpersonal communication can hinder socially adequate reactions and may be associated with fewer enduring social relationships. The link between impaired complex emotion recognition and real-life outcomes and the fact that prior evidence shows that social support is an important factor for successful abstinence (18) suggest that treatments incorporating social cognitive remediation strategies specifically targeting complex emotion recognition could ameliorate therapy and real-life outcomes.

Future research should strive to examine if social cognitive treatment approaches constitute an effective addition to conventional therapy. Moreover, longitudinal and prospective studies could shed more light on the causal relationship of cocaine use and prosodic and cross-modal information processing deficits. Lastly, functional neuroimaging may provide valuable information if there is a biological correlate to drug use patterns differing in severity and associated complex emotion recognition deficits.

## Conflict of Interest Statement

The authors declare that the research was conducted in the absence of any commercial or financial relationships that could be construed as a potential conflict of interest.

## Appendix

### Methods

#### Methodology of the urine analysis

Urine toxicology analyses comprised the compounds/substances tetrahydrocannabinol, cocaine, amphetamines, benzodiazepines, opioids, and methadone and were assessed by a semi-quantitative Enzyme Multiplied Immunoassay method (Dimension RXL Max, Siemens, Erlangen, Germany) (54).

#### Methodology of the hair analysis

If participants’ hair was long enough, one sample of 6 cm hair (from the scalp) was taken and subsequently divided into two subsamples of 3 cm length. The following compounds were assessed: cocaine, benzoylecgonine, ethylcocaine, norcocaine, amphetamine, methamphetamine, MDMA, MDEA, MDA, morphine, codeine, methadone EDDP (primary methadone metabolite), tramadol, and methylphenidate.

For our routine protocol for drugs of abuse analysis a three step washing procedure with water (2 min shaking, 15 ml), acetone (2 min, 10 ml), and finally hexane (2 min, 10 ml) of hair was performed. Then the hair samples were dried at ambient temperatures, cut into small snippets and extracted in two steps, first with methanol (5 ml, 16 h ultrasonication) and a second step with 3 ml MeOH acidified with 50 μL hydrochloric acid 33% (3 h, ultrasonication). The extracts were dried and the residue reconstituted with 50 μL MeOH and 500 μL 0.2 mM ammonium formate (analytical grade) in water. As internal standards deuterated standards of the following compounds were used, added as mixture of the following compounds: cocaine-d3, benzoylecgonine-d3, ethylcocaine-d3, morphine-d3, MAM-d3, codeine-d3, dihydrocodeine-d3, amphetamine-d6, methamphetamine-d9, MDMA-d5. MDEA-d6, MDA-d5, methadone-d9, EDDP-d3, methylphenidate-d9, tramadol-d3, oxycodone-d3, and ephedrine-d3. All deuterated standards were from ReseaChem (Burgdorf, Switzerland), the solvents for washing and extraction were of analysis grade and obtained from Merck (Darmstadt, Germany); LC-solvents were of HPLC grade and were obtained from Sigma Aldrich (Buchs, Switzerland).

The LC-MS/MS apparatus was an ABSciex QTrap 3200 [Analyst software Version 1.5, Turbo V ion source operated in the ESI mode, gas 1, nitrogen (50 psi); gas 2, nitrogen (60 psi); ion spray voltage, 3500 V; ion source temperature, 450°C; curtain gas, nitrogen (20 psi) collision gas, medium], with a Shimadzu Prominence LC-system (Shimadzu CBM 20 A controller, two Shimadzu LC 20 AD pumps including a degasser, a Shimadzu SIL 20 AC autosampler, and a Shimadzu CTO 20 AC column oven, Shimadzu, Duisburg, Germany). Gradient elution was performed on a separation column (Synergi 4 μ POLAR-RP 80A, 150 × 2.0 with a POLAR-RP 4 × 2.0 Security Guard Cartridge (Phenomenex, Aschaffenburg, Germany). The mobile phase consisted of 1 mM ammonium formate buffer adjusted to pH 3.5 with formic acid (eluent A) and acetonitrile containing 1 mM ammonium formate and 1 mM formic acid (eluent B). The Analysis was performed in MRM mode with two transitions per analyte and one transition for each deuterated internal standard, respectively.
